# Salidroside ameliorates acute liver transplantation rejection in rats by inhibiting neutrophil extracellular trap formation: Salidroside ameliorates AR of liver transplantation

**DOI:** 10.3724/abbs.2024055

**Published:** 2024-05-07

**Authors:** Xiaoyan Qin, Han Wang, Qi Li, Dingheng Hu, Liangxu Wang, Baoyong Zhou, Rui Liao, Yanyao Liu

**Affiliations:** 1 Department of Hepatobiliary Surgery the First Affiliated Hospital of Chongqing Medical University Chongqing 400042 China; 2 Department of General Surgery and Trauma Surgery Children’s Hospital of Chongqing Medical University National Clinical Research Center for Child Health and Disorders Ministry of Education Key Laboratory of Child Development and Disorders Chongqing Key Laboratory of Structural Birth Defect and Reconstruction Chongqing 400014 China

**Keywords:** salidroside, neutrophil extracellular traps, acute rejection, liver transplantation, HMGB1

## Abstract

Acute rejection is an important factor affecting the survival of recipients after liver transplantation. Salidroside has various properties, including anti-inflammatory, antioxidant, and hepatoprotective properties. This study aims to investigate whether salidroside can prevent acute rejection after liver transplantation and to examine the underlying mechanisms involved. An *in vivo* acute rejection model is established in rats that are pretreated with tacrolimus (1 mg/kg/d) or salidroside (10 or 20 mg/kg/d) for seven days after liver transplantation. In addition, an *in vitro* experiment is performed using neutrophils incubated with salidroside (1, 10, 50 or 100 μM). Hematoxylin-eosin staining, terminal deoxynucleotidyl transferase dUTP nick-end labeling staining, immunosorbent assays, immunofluorescence analysis, Evans blue staining, and western blot analysis are performed to examine the impact of salidroside on NET formation and acute rejection *in vitro* and *in vivo*. We find that Salidroside treatment reduces pathological liver damage, serum aminotransferase level, and serum levels of IL-1β, IL-6, and TNF-α *in vivo*. The expressions of proteins associated with the HMGB1/TLR-4/MAPK signaling pathway (HMGB1, TLR-4, p-ERK1/2, p-JNK, p-P38, cleaved caspase-3, cleaved caspase-9, Bcl-2, Bax, IL-1β, TNF-α, and IL-6) are also decreased after salidroside treatment. *In vitro* experiments show that the release of HMGB1/TLR-4/MAPK signaling pathway-associated proteins from neutrophils treated with lipopolysaccharide is decreased by salidroside. Moreover, salidroside inhibits NETosis and protects against acute rejection by regulating the HMGB1/TLR-4/MAPK signaling pathway. Furthermore, salidroside combined with tacrolimus has a better effect than either of the other treatments alone. In summary, salidroside can prevent acute liver rejection after liver transplantation by reducing neutrophil extracellular trap development through the HMGB1/TLR-4/MAPK signaling pathway.

## Introduction

Liver transplantation is the mainstay treatment for end-stage liver disease and acute liver failure [1]. Acute rejection (AR), a common cause of poor prognosis after liver transplantation, occurs in approximately 20%‒30% of patients within the first 12 months [2]. Although immunosuppressive protocols have improved the prognosis of patients after liver transplantation, some studies have shown that immunosuppressive drugs have some adverse effects, including metabolic disorders, severe infection, and tumor recurrence, which limits their application in liver transplantation [ 3, 4]. Therefore, more studies are required to elucidate AR pathogenesis to establish novel therapeutic targets for AR.

The nuclear protein high-mobility group box-1 (HMGB1) is highly conserved and is an immunomodulatory factor involved in hepatic ischemia-reperfusion injury (IRI) and acute rejection after liver transplantation [ 5, 6]. Recent studies have revealed that the HMGB1-TLR-4 signaling pathway promotes the pathophysiology of liver IRI by activating the inflammatory response. Moreover, HMGB1 can induce acute rejection after liver transplantation by activating dendritic cells [ 7, 8]. Many studies have shown that HMGB1 participates in the pathological processes of many inflammation-related diseases by regulating neutrophil activation and NET formation [ 9, 10].

Neutrophil extracellular traps are extracellular DNA fibers decorated with histones and granular proteins [11]. NETs can neutralize and kill bacteria. However, NET dysregulation may also induce immune-related adverse events, such as hepatic IRI and acute liver rejection after liver transplantation [ 12, 13]. Numerous investigations have demonstrated that NETosis exacerbates inflammation and contributes to brain ischemia through HMGB1 signaling. Necrotic hepatocytes induce NET formation and exacerbate hepatic IRI by releasing HMGB1 [ 14, 15]. An earlier study demonstrated that excessive neutrophil accumulation, neutrophil hyperreactivity, and the uncontrolled formation of neutrophil extracellular traps (NETs) after liver transplantation promote the creation of a local liver inflammatory microenvironment and acute rejection after liver transplantation [ 16, 17]. These studies indicate that HMGB1-induced NET formation promotes the incidence and development of acute rejection after liver transplantation.

Salidroside, which is extracted from various Rhodiola plants, can treat ischemic stroke, Alzheimer’s disease, and cardiovascular diseases [ 18, 19]. Salidroside has broad pharmacological effects, including inhibiting hypoxia, inflammation, and oxidation [20]. Moreover, salidroside can treat hepatic and brain IRI [ 21, 22]. However, studies have not explored the effects of salidroside on NET formation or its role in alleviating acute rejection following liver transplantation.

In this study, we established a liver transplantation rat model to examine the effect of salidroside on acute rejection after liver transplantation by targeting HMGB1 and NETosis. The effect of the combination of salidroside and tacrolimus on acute rejection following liver transplantation was also evaluated.

## Materials and Methods

### Ethics statement

This study was approved by the Ethics Committee of Animal and Human Experimentation of Chongqing Medical University. All experiments adhered to the Declaration of Helsinki. Efforts were made to minimize animal suffering and discomfort, and the fewest number of animals were used in accordance with the 3Rs principle.

### Animals and liver transplantation

**Figure 1:**
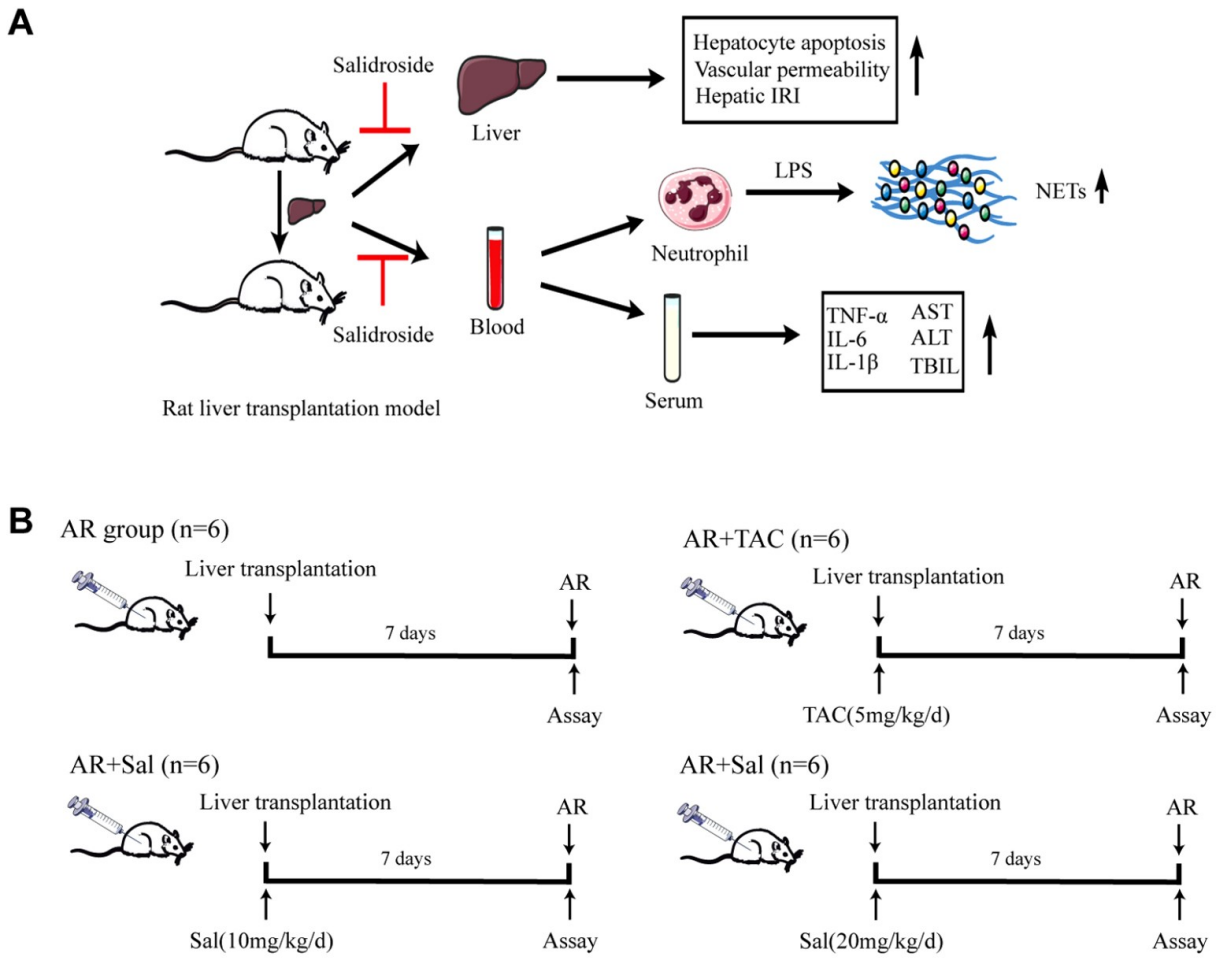
Figure 1 The experimental design (A) Flow chart of the experimental design of the animal experiment. (B) Schematic diagram showing the grouping of the rats. The rats were divided into 6 groups. The sham group received an abdominal incision, exposing the hepatic portal vein (n=6); the AR group received no treatment after liver transplantation; the AR+TAC group received intraperitoneal administration of TAC (1 mg/kg/d) for 7 days after liver transplantation; the AR+Sal group received intraperitoneal administration of salidroside (10 or 20 mg/kg/d) for 7 days after liver transplantation; and the AR+TAC+Sal group received intraperitoneal administration of TAC (1 mg/kg/d) and salidroside (20 mg/kg/d) for 7 days after liver transplantation.

Inbred male Brown Norway (BN) rats and Lewis (LEW) rats (SPF grade, 250‒280 g) were acquired from the Chongqing Medical University Experimental Animal Center (Chongqing, China). Orthotopic liver transplantation was conducted with a magnetic anastomosis approach as described by Yang *et al*. [23] with details shown in Figure 1A. The rats were randomly divided into 6 groups (with 12 rats per group): (1) rats in the Sham group underwent surgery via an abdominal incision to expose the hepatic portal vein; (2) the rats in the acute rejection (AR) group did not receive any treatment after liver transplantation; (3) in the AR+TAC group, the rats were given intraperitoneal injections of TAC (1 mg/kg/d, MedChemExpress, New Jersey, USA) for 7 days following liver transplantation; (4 and 5) the AR+Sal group received intraperitoneal injections of salidroside (10 or 20 mg/kg/d; YuanyeBio, Shanghai, China) for 7 days after liver transplantation; (6) the AR+TAC+Sal group was treated with both TAC (1 mg/kg/d) and salidroside (20 mg/kg/d) for 7 days after liver transplantation. At the end of the experiment, the rats were euthanized, and their liver tissues and serum were collected and stored at ‒80°C.

### Isolating serum and rat primary neutrophils

Following euthanasia, blood samples were collected from the heart. The samples were then centrifuged at 3000 *g* for 10 min at 4°C, and the resulting supernatant was stored at ‒80°C for further biochemical analysis. Neutrophils were isolated from peripheral blood using a peripheral blood neutrophil isolation kit (TBD, Tianjin, China) according to the manufacturer’s instructions.

### Histology and TUNEL analysis

Liver portions were preserved with 4% paraformaldehyde, stained with hematoxylin and eosin (HE) (Beyotime, Shanghai, China), and then cut into sections. The RAI score according to the Banff criterion [24], which includes portal inflammation, bile duct inflammation damage and venous endothelial inflammation, was used to measure hepatic pathological impairment based on the HE results. Hepatic apoptosis was assessed with a TUNEL kit (Beyotime) in accordance with the manufacturer’s instructions.

### Analysis of serum cytokines and liver enzymes

Serum IL-1β, IL-18, and TNF-α levels were determined with an enzyme-linked immunosorbent assay (ELISA) kit (Neobioscience, Beijing, China) according to the manufacturer’s guidelines to measure the release of inflammatory cytokines. A liver enzyme kit (Jiancheng Bioengineering Institute, Nanjing, China) was used to detect the serum levels of aspartate aminotransferase (AST) and alanine aminotransferase (ALT) according to the manufacturer’s protocol.

### Quantification of extracellular DNA

The Quant-it PicoGreen dsDNA test kit (Life Technologist, New York, USA) was used to measure extracellular DNA/NETs in neutrophils. A microplate reader (Thermo Fisher Scientific, Waltham, USA) was used to determine the fluorescence signal intensity at an excitation wavelength of 485 nm and an emission wavelength of 535 nm.

### Cell viability assay

Neutrophils were cultured in a 96-well plate (1×10 ^4^ cells per well) and treated with lipopolysaccharide (0.1, 1, 10, or 25 μg/μL) for 24 h. The cells in each well were treated with 20 μL of MTT (5 mg/mL; Sigma-Aldrich, St.Louis, USA) solution for 4 h. The culture medium was removed, and 150 μL of dimethyl sulfoxide (DMSO) was added to each well. Finally, the microplate reader was used to measure the absorption value of each well at a wavelength of 490 nm. The average absorbance relative to that of the control group was calculated.

### Immunofluorescence assay

To stain the cells, primary antibodies targeting citrullinated histone-3 (H3cit) (Abcam, Cambridge, UK) and myeloperoxidase (MPO) (Abcam) were used to stain enzymes associated with NETs. DNA and NETs were then stained with 4,6-diamidino-2-phenylindole (DAPI) (Beyotime). The cells were diluted in DAPI solution at a 1:1000 ratio and incubated at room temperature for 3 min. After two times wash with PBS, the cells were coated with an anti-fluorescence quencher (Beyotime). The localization and structure of NETs were examined using a confocal fluorescence microscope (Olympus, Tokyo, Japan).

### Western blot analysis

RIPA buffer was used to extract total protein from the cells. For total liver proteins, a radioimmunoprecipitation assay solution with a proteinase inhibitor cocktail was used for lysis. The proteins were separated by 10% SDS-PAGE and transferred to polyvinylidene fluoride membranes (Millipore, Billerica, USA). The membranes were incubated overnight at 4°C with primary antibodies ( Table 1), followed by incubation with horseradish peroxidase-conjugated secondary antibodies (A0192, 1:1000; Beyotime) at room temperature for 1 h. Signal detection was performed using a gel imaging device and a chemiluminescent reagent (ChemiScope 2850; Clinx Science, Shanghai, China). The immunoreactive bands were quantified using ImageJ software.

**Table:** **Table 1** Antibodies used for immunofluorescence staining and western blot analysis

Target	Dilution	Supplier	Code
TLR-4	WB (1/300)	Abcam	ab217274
HMGB1	IF (1/500), WB (1/1000)	CST	6893
MPO	IF (1/100)	Abcam	ab208670
p-ERK1/2	WB (1/1000)	CST	4370
ERK1/2	WB (1/1000)	CST	4695
p-JNK	WB (1/1000)	CST	4668
JNK	WB (1/1000)	CST	9252
p-P38	WB (1/1000)	CST	4511
P38	WB (1/1000)	CST	8690
IL-1β	WB (1/1000)	Beyotime	AG2258
TNF-α	WB (1/1000)	Beyotime	AF2808
IL-6	WB (1/1000)	Beyotime	AF0201
β-actin	WB (1/1000)	Beyotime	AF5003

IF, immunofluorescence staining; WB, western blot analysis.

### Vascular permeability assay

The mice were anesthetized using isoflurane and then treated with Evans blue (40 mg/kg). After 1 h, the rats were sacrificed, and 100 mg of liver tissue was collected. The samples were incubated in 3 mL of formamide at 50°C overnight, after which the liver supernatants were collected via centrifugation (400 *g* for 5 min at 4°C). The microplate reader was used to measure the fluorescence at 620 nm and 740 nm.

### Statistical analysis

Data are presented as the mean±standard deviation. Statistical analysis was performed using Student’s *t* test for two groups and one-way ANOVA for comparisons involving multiple groups. All analyses were performed with GraphPad Prism v8.0. Statistical difference was considered significant at *P*<0.05.

## Results

### Salidroside suppresses NET formation and alleviates acute rejection in rats after liver transplantation

**Figure 2:**
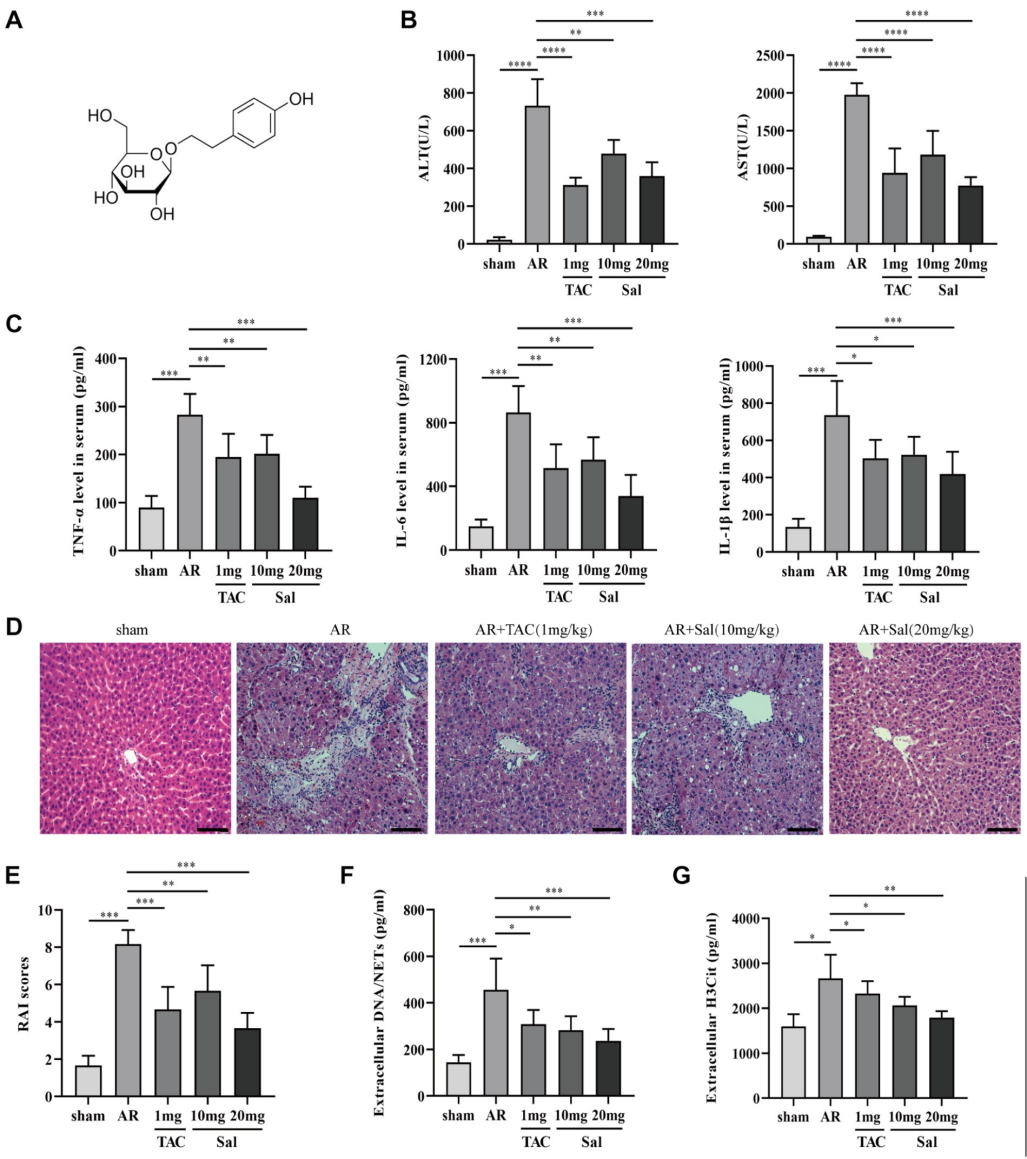
Figure 2 Salidroside inhibits NET formation and alleviates acute rejection after liver transplantation (A) Chemical structure of salidroside. (B) Serum levels of ALT and AST in the different groups. (C) Serum levels of IL-1β, IL-6, and TNF-α in the different groups. (D) Representative images of the hepatic histopathological changes in the livers of rats observed after HE staining (magnification, ×200; scale bar: 200 μm; n=6). (E) RAI scores of different groups according to the Banff scheme. (F) NET production in the serum of rats in different groups. (G) Increased levels of circulating H3Cit in the serum of rats in different groups. The significant differences among groups were compared using one-way ANOVA. *P<0.05, **P<0.01, ***P<0.001.

The degree of acute rejection following liver transplantation increased with time and peaked within 7 days, which is consistent with previous study [17]. Notably, the rat models were examined 7 days after transplantation. The results showed that salidroside ( Figure 2A) significantly decreased liver cell damage in rats ( Figure 2B). The serum levels of IL-1β, IL-6, and TNF-α were significantly elevated in the AR group ( Figure 2C). The administration of 10 or 20 mg/kg/d salidroside resulted in a gradual reduction in the levels of cytokines compared with those in the AR group. Histological examination by HE staining revealed severe and progressive AR in liver tissues. The liver is characterized by damage to the bile ducts, extensive hepatocyte necrosis, and leukocyte infiltration. However, treatment with salidroside (10 or 20 mg/kg/d) effectively alleviated the pathological alterations in the liver. These results were further supported by evaluating the rejection activity index (RAI) score based on the Banff schema, as shown in Figure 2D,E. Compared with the sham group, the AR group exhibited elevated serum levels of extracellular DNA/NETs and H3Cit. Salidroside treatment (10 or 20 mg/kg/d) significantly decreased the serum levels of extracellular DNA/NETs and H3Cit ( Figure 2F,G). These data indicated that salidroside suppresses NET formation and alleviates acute rejection after liver transplantation.

### Salidroside inhibits hepatocyte apoptosis in rats after liver transplantation

**Figure 3:**
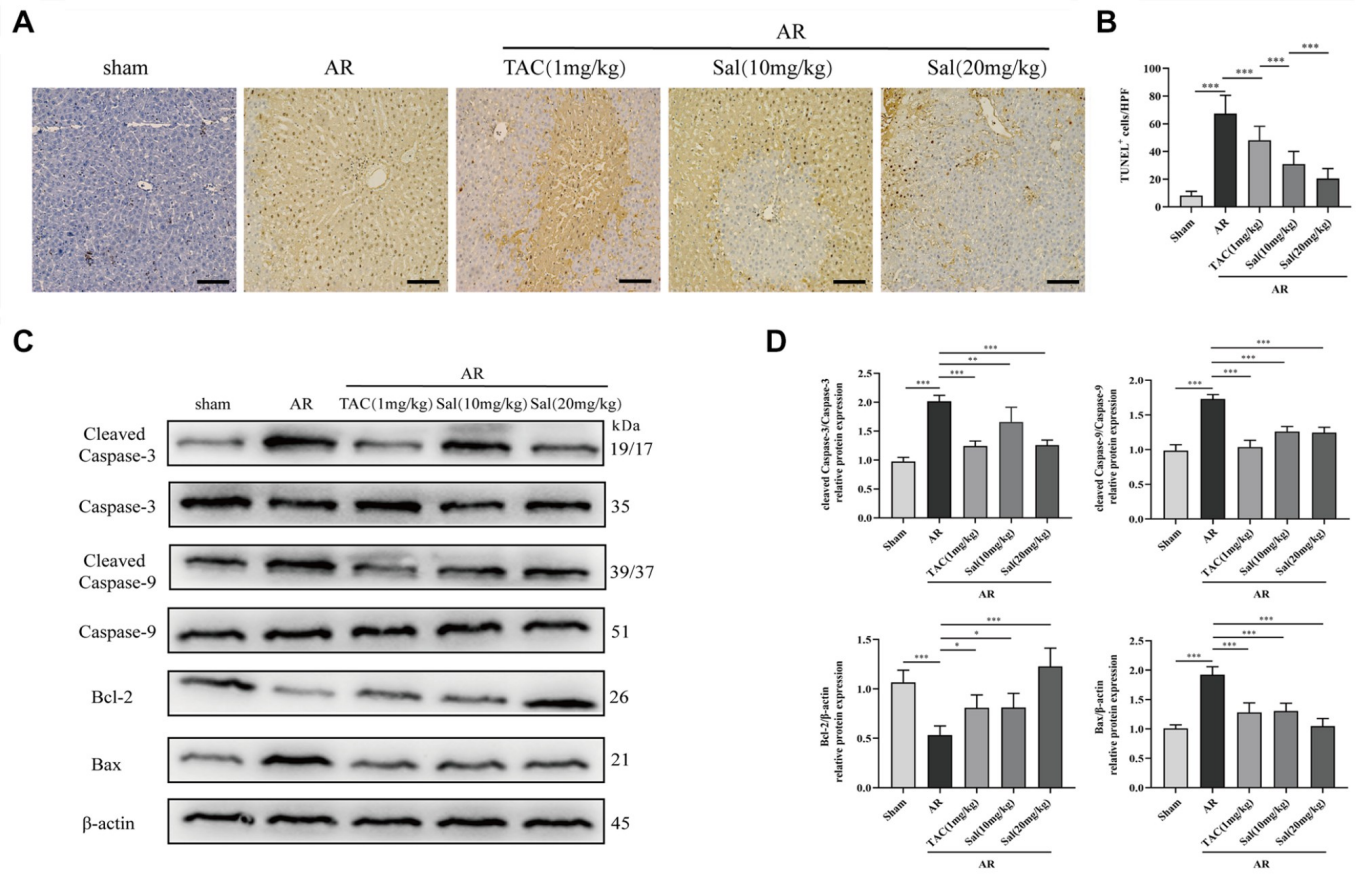
Figure 3 Salidroside inhibits hepatocyte apoptosis in rats after liver transplantation (A) Hepatic apoptosis detected by TUNEL in different groups (magnification, ×200; n=6). (B) TUNEL+ cells of the liver based on the TUNEL assay. (C) Representative western blots showing the levels of apoptosis-associated proteins in the different groups. (D) The quantitative results of the western blots. Statistical differences among groups were determined using one-way ANOVA. *P<0.05, **P<0.01, ***P<0.001. ns, not significant.

A TUNEL kit and western blot analysis were used to evaluate the degree of hepatocyte apoptosis following salidroside treatment *in vivo*. There were fewer TUNEL-positive cells in the sham group than in the AR group. However, salidroside treatment (10 mg/kg/d or 20 mg/kg/d) significantly decreased the number of TUNEL-positive cells in a dose-dependent manner ( Figure 3A,B). The expressions of apoptosis-associated proteins, including cleaved caspase-3, cleaved caspase-9, and Bax, in liver tissues were markedly greater in the AR group than in the Sham group. In contrast, Bcl-2 expression was significantly downregulated in the AR group compared with that in the sham group. Salidroside treatment (10 or 20 mg/kg/d) downregulated cleaved caspase-3, cleaved caspase-9, and Bax expression levels but increased Bcl-2 expression level ( Figure 3C,D). These findings revealed that salidroside protects against acute rejection after liver transplantation by inhibiting hepatocellular apoptosis.

### Salidroside suppresses liver inflammation in rats after liver transplantation via HMGB1-related signaling pathways

**Figure 4:**
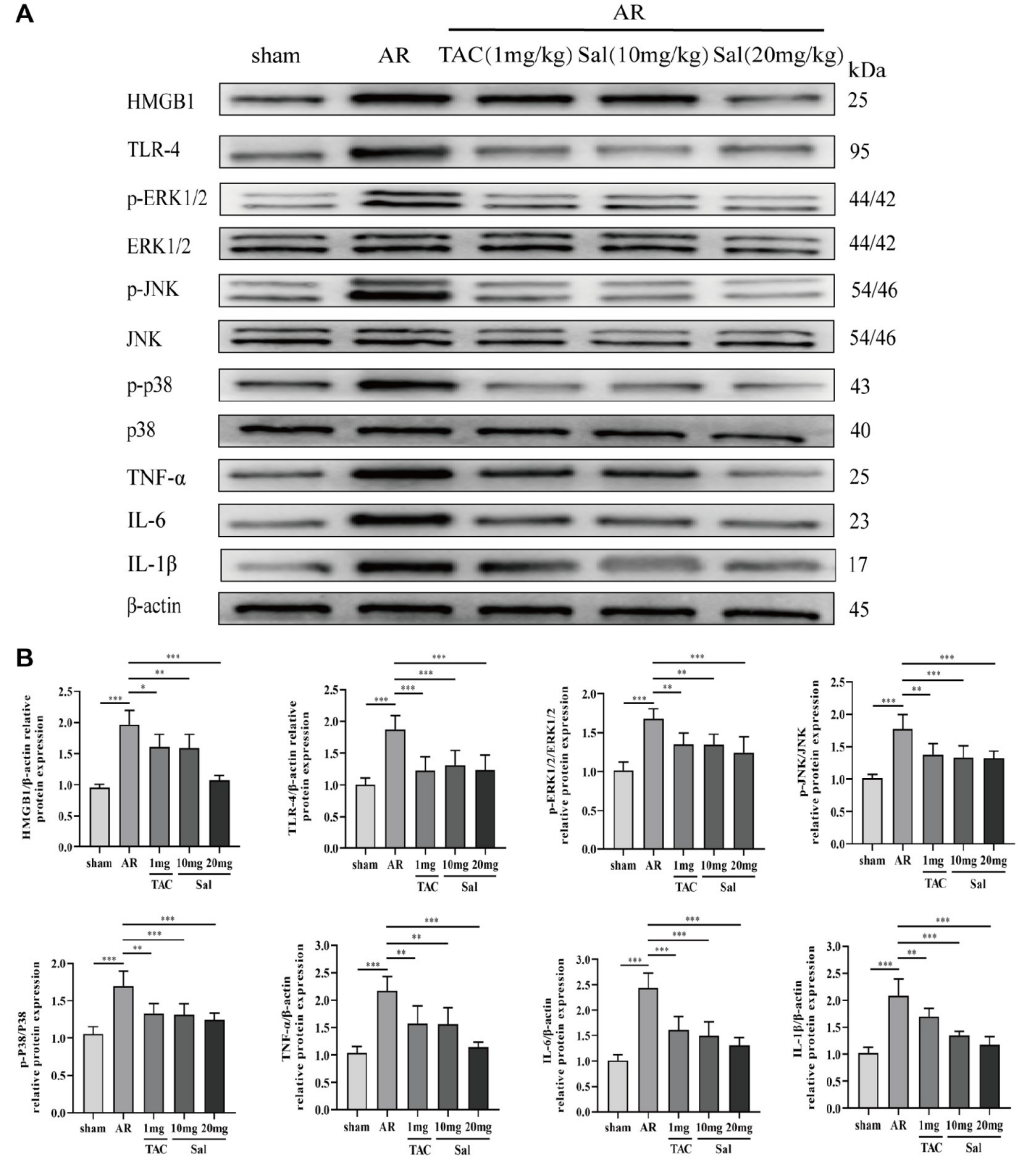
Figure 4 Salidroside suppresses liver inflammation via the HMGB1 signaling pathway after liver transplantation (A) Representative western blots showing HMGB1/TLR-4/MAPK signaling pathway-related proteins in the different groups. (B) Quantitative analysis of the western blots. The significant differences among groups were compared using one-way ANOVA. *P<0.05, **P<0.01, ***P<0.001.

HMGB1, TLR-4, p-ERK1/2, p-JNK, p-P38, IL-1β, IL-6, and TNF-α expressions in liver tissue were significantly greater in the AR group than in the sham group. Salidroside therapy (10 or 20 mg/kg/d) gradually downregulated the expressions of HMGB1/TLR-4/MAPK signaling pathway-related proteins and proinflammatory proteins ( Figure 4A,B). These data indicated that salidroside suppresses liver inflammation after liver transplantation via HMGB1-related signaling pathways.

### Salidroside improves LPS-induced NET formation

**Figure 5:**
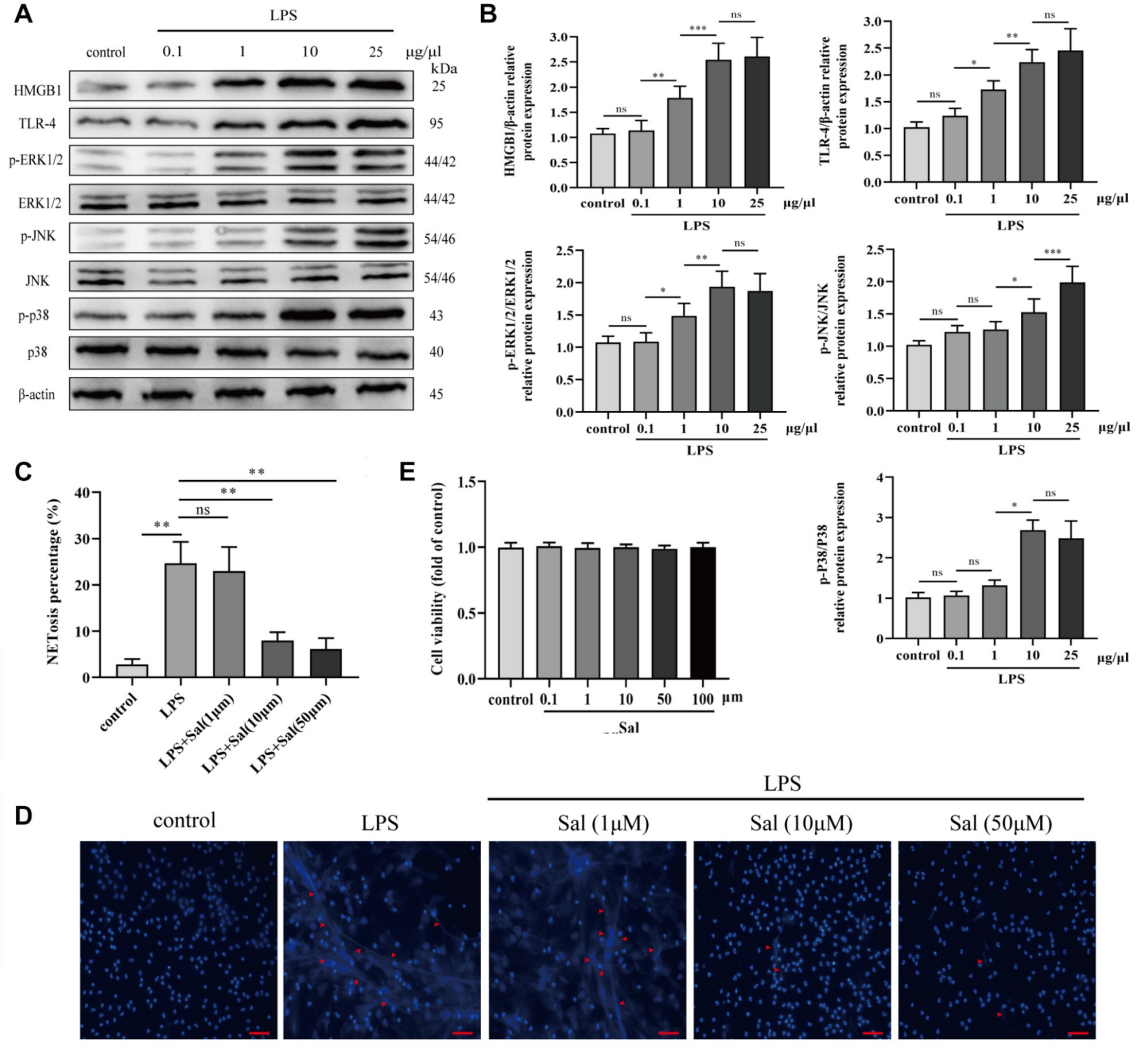
Figure 5 Salidroside improves LPS-induced NET formation (A) The expression levels of HMGB1/TLR-4/MAPK signaling pathway-associated proteins were detected by western blot analysis. (B) Quantitative analysis of the western blots. (C,D) The separated neutrophils were pretreated with or without Sal (1 μM, 10 μM, or 50 μM) for 60 min and then activated with 25 μg/μL LPS for 1 h. Neutrophils in the control group received no treatment. The structure of the NETs (red arrowheads) was observed using laser scanning confocal microscopy (magnification, ×200; scale bar: 200 μm), and quantitative NET formation by neutrophils was analyzed. (E) Neutrophils were incubated with different concentrations of lipopolysaccharide (0.1–25 μL/μg) for 24 h. Cell viability was determined using MTT. Statistical differences among groups were compared using one-way ANOVA. *P<0.05, **P<0.01, ***P<0.001. ns, not significant.

An *in vitro* experiment was performed to assess cell survival and the inhibition of NET formation by salidroside in lipopolysaccharide (LPS)-stimulated neutrophils. LPS stimulation (0.1-25 g/L) upregulated HMGB1, TLR-4, p-ERK1/2, p-JNK, and p-P38 levels, indicating that the HMGB1/TLR-4/MAPK signaling pathway may participate in LPS-induced NET formation ( Figure 5A,B). Neutrophils were then pretreated with various concentrations of salidroside (1 μM, 10 μM, or 50 μM) for 1 h before LPS stimulation to investigate the inhibitory effects of salidroside on NET formation. NET expression was quantified based on the fluorescence intensity. Salidroside significantly inhibited NET formation ( Figure 5C,D). Moreover, 100 μM salidroside did not induce toxicity in neutrophils ( Figure 5E). These data indicated that salidroside improves LPS-induced NET formation.

### Salidroside suppresses LPS-induced NET formation by blocking the activation of the HMGB1 signaling pathway

**Figure 6:**
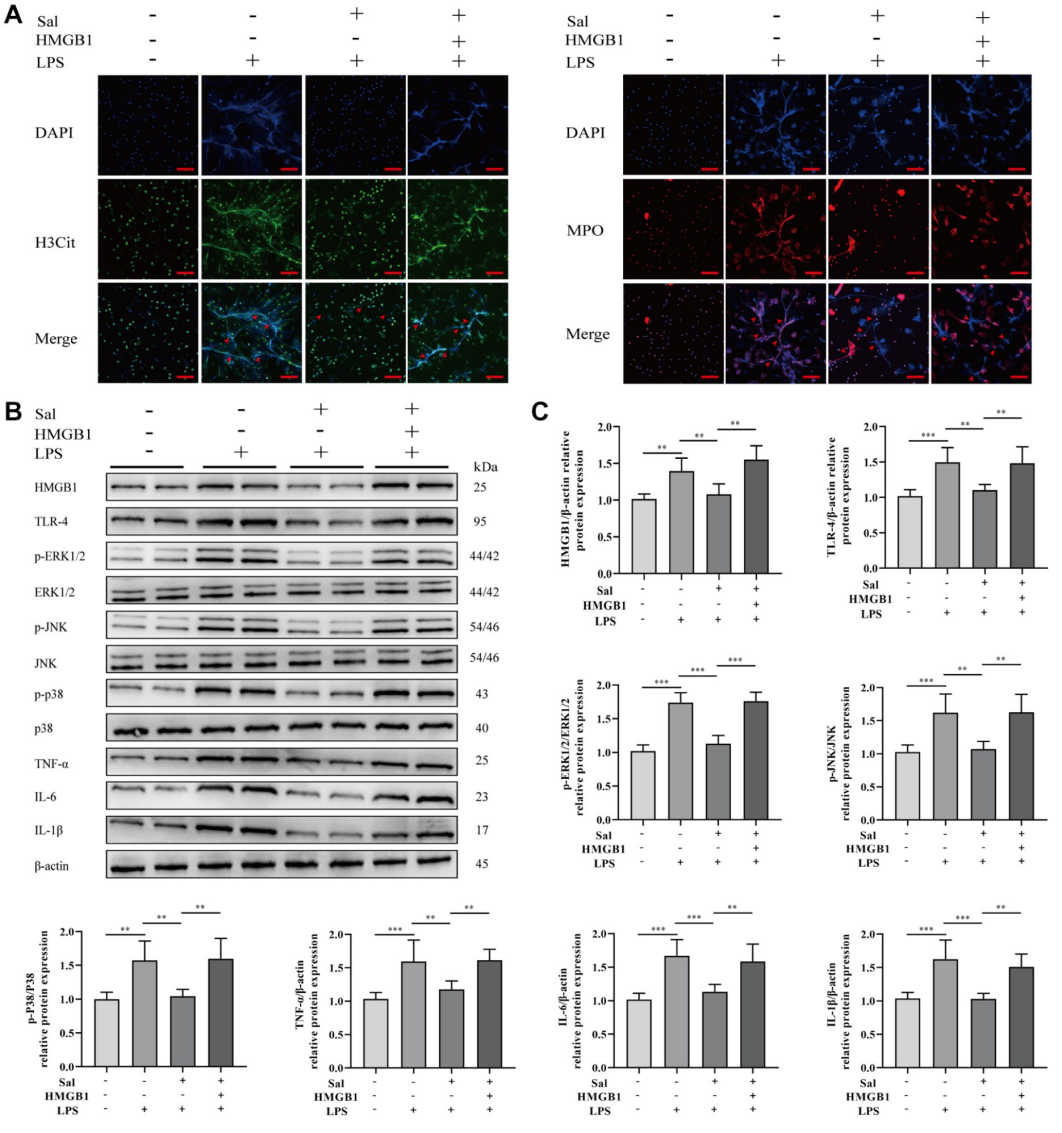
Figure 6 Salidroside suppresses LPS-induced NET formation by inhibiting the activation of the HMGB1 signaling pathway (A) Immunofluorescence analysis of neutrophil extracellular trap formation induced by Sal and HMGB1. (B) Representative western blots showing proteins associated with the HMGB1/TLR-4/MAPK signaling pathway. (C) Quantitative analysis of the western blots. Differences among groups were compared using one-way ANOVA. **P<0.01, ***P<0.001.

Immunofluorescence staining was used to examine the effect of HMGB1 and salidroside on NET formation. LPS stimulation enhanced NET formation, while salidroside decreased NET formation. However, HMGB1 partly reversed the effects of salidroside ( Figure 6A). Western blot analysis results indicated that salidroside treatment significantly suppressed HMGB1/TLR-4/MAPK signaling pathway activation compared with that in the LPS group. In contrast, HMGB1 pretreatment significantly upregulated the expressions of proteins associated with the HMGB1/TLR-4/MAPK pathway and partially reversed the effects of salidroside ( Figure 6B,C). These findings suggested that salidroside inhibits LPS-induced NET formation by suppressing the activation of the HMGB1/TLR-4/MAPK signaling pathway and the production of inflammatory cytokines.

### Synergistic effects of salidroside and tacrolimus on acute rejection in rats after liver transplantation

**Figure 7:**
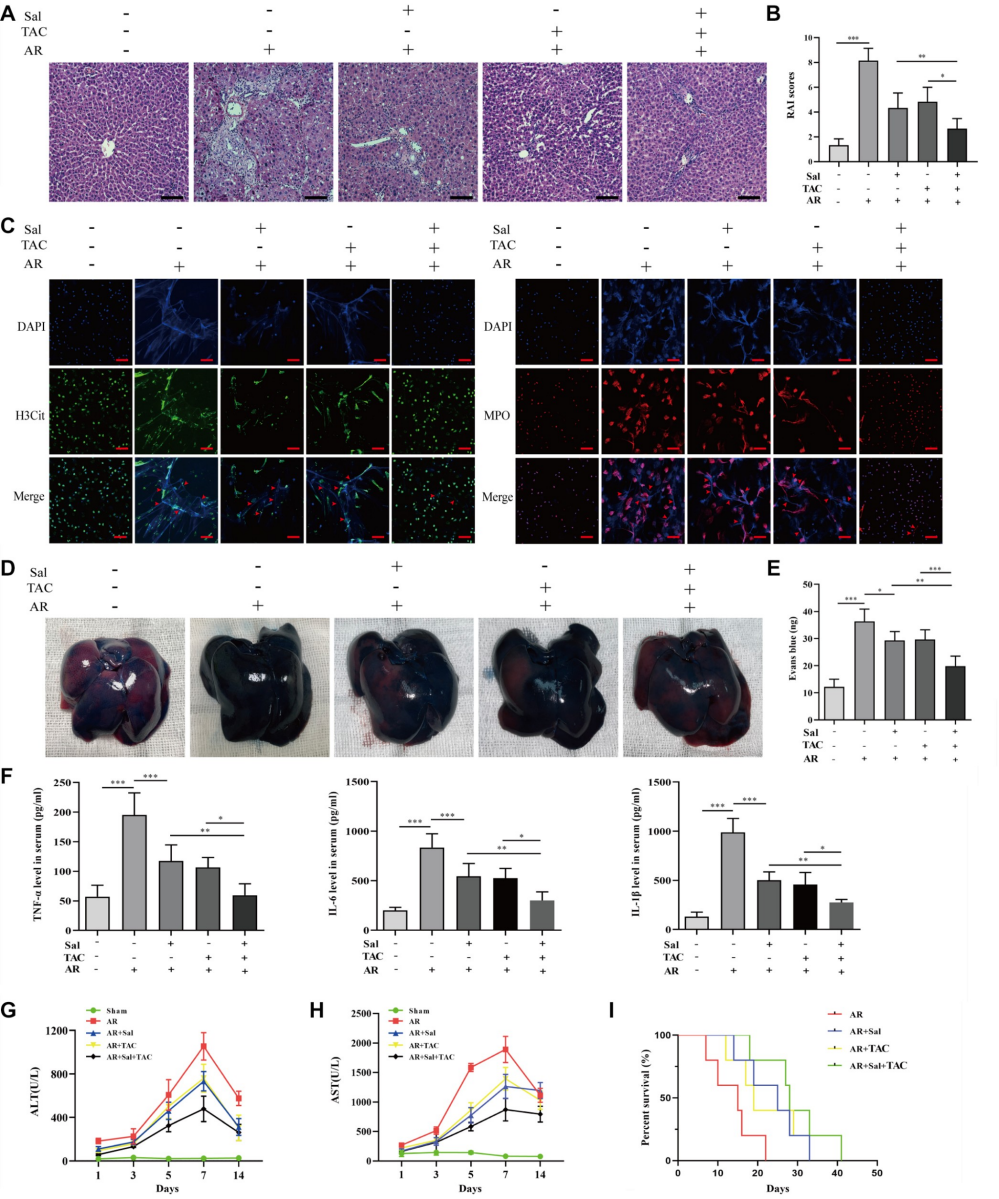
Figure 7 Synergistic effects of salidroside and tacrolimus on acute rejection after liver transplantation (A, B) Pathological liver alterations after salidroside and tacrolimus administration. (C) Immunofluorescence analysis of neutrophil extracellular trap formation after salidroside and tacrolimus treatment. (D,E) Liver vascular leakage analysis. (F) Serum levels of TNF-α, IL-6, and IL-1β in the different groups. (G) Serum levels of ALT on days 1, 3, 5, 7, and 14 after liver transplantation. (H) Serum levels of AST on days 1, 3, 5, 7, and 14 after liver transplantation. (I) Survival analysis of rats in different groups. Differences among groups were compared using one-way ANOVA. *P<0.05, **P<0.01, ***P<0.001.

HE staining was performed to determine the effect of salidroside and tacrolimus on the AR group. The results showed that the RAI scores were significantly lower in the AR+Sal and AR+TAC groups than in the AR group. Salidroside and tacrolimus significantly reduced the severity of the histological alterations in the liver compared with those in the AR group ( Figure 7A,B). The effects of salidroside and tacrolimus on NET formation, liver vascular permeability changes, and proinflammatory factor release in rats after liver transplantation were investigated by immunofluorescence assays, Evans blue assays, and ELISA. Salidroside and tacrolimus slightly reduced the formation of NETs by neutrophils, microvascular leakage in liver tissues, and the levels of inflammatory factors in the serum ( Figure 7C‒F). Moreover, the hepatic aminotransferase levels in the serum were slightly reduced after treatment with salidroside and tacrolimus, which is consistent with the findings of previous studies ( Figure 7G,H). Importantly, treatment with salidroside and tacrolimus partially improved survival outcomes compared with those in the AR group. The combination of salidroside and tacrolimus had better effects on liver injury, NET formation, microvascular leakage, inflammatory factor secretion, and survival outcomes in rats than either drug alone. These findings suggest that combining salidroside and tacrolimus can significantly alleviate acute rejection after liver transplantation compared with either of the two treatments alone.

## Discussion

Liver transplantation is the mainstay treatment strategy for end-stage liver diseases [25]. Acute rejection after liver transplantation significantly affects the prognosis of patients with end-stage liver diseases [26]. A previous study demonstrated that massive infiltration of neutrophils and NET formation in the donor liver promote the development of an inflammatory microenvironment within hepatic tissue, leading to acute rejection [ 16, 17]. Other investigations reported that inhibiting inflammatory responses by targeting proinflammatory molecules, such as HMGB1, can mitigate acute rejection and alleviate liver injury [ 6, 8]. Therefore, pharmaceutical therapies targeting specific molecules and cells can treat or prevent acute rejection after liver transplantation. Salidroside improves hepatic dysfunction, decreases inflammatory cytokines, inhibits histopathological changes, and decreases NET formation. In addition to reducing hepatocyte apoptosis, salidroside also suppresses LPS-induced NET formation by inhibiting the activation of the HMGB1/TLR-4/MAPK signaling pathway. Moreover, the combination of salidroside and tacrolimus significantly alleviates acute rejection after liver transplantation compared with a single administration of either treatment. Therefore, this study is expected to provide ideas for the clinical prevention and treatment of acute rejection after liver transplantation.

Salidroside, which is derived from Rhodiola plants, possesses a range of biological activities, such as anti-inflammatory and antioxidant properties [ 27, 28]. Hu *et al*. [29] showed that salidroside protects against metabolic stress-induced nonalcoholic steatohepatitis (NASH) progression by activating AMPK signaling. Feng *et al*. [30] also showed that salidroside prevents IRI in hepatocytes by reducing MAPK signaling activity. Moreover, it suppresses hepatic IRI when used as a pretreatment during liver transplantation. Salidroside can also protect hepatocytes against apoptosis by activating the GSK-3/Nrf2-dependent antioxidant response [31]. Salidroside reduces the serum levels of inflammatory cytokines, such as IL-1β, IL-6, and TNF-α, and suppresses acute rejection-induced hepatocellular damage by decreasing the serum alanine transaminase (ALT) and aspartate transaminase (AST) levels, histological characteristics of liver injury, and apoptosis. Moreover, the formation of NETs is reduced, and the serum levels of extracellular DNA/NETs and H3Cit are decreased by salidroside treatment. A previous study showed that NETs promotes the development of sterile inflammation associated with acute rejection, indicating that NETs are promising therapeutic targets for the prevention of acute rejection after liver transplantation [17]. *In vitro* experiments revealed that salidroside improves LPS-induced NET production via the HMGB1/TLR-4/MAPK signaling pathway without causing any toxicity. Similarly, *in vivo* experiments demonstrated that salidroside treatment decreases NET production via the HMGB1/TLR4/MAPK signaling pathway, thereby improving hepatic dysfunction, decreasing inflammatory cytokines, and attenuating hepatic histopathological changes.

HMGB1, which is a protein that is found in large quantities in chromosomes, acts as a damage-associated molecular pattern that initiates innate inflammatory responses against infection and injury [32]. HMGB1 contributes to the pathogenesis of multiple liver diseases, such as hepatic IRI and acute liver rejection, through TLRs [33]. Moreover, recombinant HMGB1 protein triggers NET formation via TLR4/9 *in vivo*. HMGB1 also promotes prothrombotic NET formation in deep venous thrombosis [ 34, 35]. Our results showed that salidroside inhibits the HMGB1/TLR-4/MAPK pathway. However, HMGB1 reverses these effects, suggesting that salidroside inhibits NETosis by preventing the activation of the HMGB1/TLR-4/MAPK signaling pathway. Tacrolimus is a widely used immunosuppressive agent. The results showed that tacrolimus alleviates acute rejection after liver transplantation and reduces NET production by inhibiting the HMGB1/TLR-4/MAPK signaling pathway. Moreover, the relationship between the mTOR signaling pathway and NET formation is consistent with the findings of previous studies [ 36, 37]. mTOR regulates NET formation, indicating that the mTOR signaling pathway participates in NET formation by regulating autophagy [38]. NETosis mainly activates the mTOR and HMGB1/TLR-4/MAPK signaling pathways. Therefore, we targeted the mTOR and HMGB1/TLR-4/MAPK signaling pathways using a combination of tacrolimus and salidroside. Individual delivery of each treatment did not completely ameliorate acute rejection after liver transplantation, whereas the combination therapy achieved near-complete remission. Nevertheless, this study has several limitations. First, we did not investigate the effectiveness and safety of salidroside treatment in patients after liver transplantation due to a lack of clinical data. Second, other properties of salidroside, such as its antioxidant activity, were not examined.

In conclusion, this study demonstrated the effect of salidroside on acute rejection after liver transplantation. Salidroside treatment improves hepatic dysfunction, inhibits histopathological changes and decreases NET formation by inhibiting the HMGB1/TLR-4/MAPK signaling pathway. Additionally, targeting NETosis-related signaling pathways may be a potential treatment strategy for acute rejection after liver transplantation.
